# Developmental Changes in Number Personification by Elementary School Children

**DOI:** 10.3389/fpsyg.2018.02214

**Published:** 2018-11-15

**Authors:** Eiko Matsuda, Yoshihiro S. Okazaki, Michiko Asano, Kazuhiko Yokosawa

**Affiliations:** ^1^Division of Advanced Information Technology and Computer Science, Tokyo University of Agriculture and Technology, Tokyo, Japan; ^2^Japan Society for the Promotion of Science (JSPS), Tokyo, Japan; ^3^Graduate School of Education, Okayama University, Okayama, Japan; ^4^Department of Psychology, College of Contemporary Psychology, Rikkyo University, Saitama, Japan; ^5^Department of Psychology, Graduate School of Humanities and Sociology, The University of Tokyo, Tokyo, Japan

**Keywords:** personification, development, synesthesia, ordinal linguistic personification, elementary school children

## Abstract

Children often personify non-living objects, such as puppets and stars. This attribution is considered a healthy phenomenon, which can simulate social exchange and enhance children's understanding of social relationships. In this study, we considered that the tendency of children to engage in personification could potentially be observed in abstract entities, such as numbers. We hypothesized that children tend to attribute personalities to numbers, which diminishes during the course of development. By consulting the methodology to measure ordinal linguistic personification (OLP), which is a type of synesthesia, we quantified the frequency with which child and adult populations engage in number personification. Questionnaires were completed by 151 non-synesthetic children (9–12 years old) and 55 non-synesthetic adults. Children showed a higher tendency than adults to engage in number personification, with respect to temporal consistency and the frequency of choosing meaningful answers. Additionally, children tended to assign unique and exclusive descriptions to each number from zero to nine. By synthesizing the series of analyses, we revealed the process in which number personification diminishes throughout development. In the discussion, we examined the possibility that number personification serves as a discrimination clue to aid children's comprehension of the relationships between numbers.

## 1. Introduction

A child's conception of personality changes throughout development. For instance, Piaget (1929) explained that children who are 5–12 years old tend to attribute consciousness to non-living matters. Younger children believe that all things are conscious; for instance, a child described a wall as if it was conscious (e.g., “a wall *feels* it is knocked down”). When they become older, they begin to think that things that can move of their own accord are conscious. For instance, a child reported that “the moon *knows* that it moves.” As they continue to grow, children start to understand that only animals are conscious. Creating friendships with imaginary beings, or “imaginary companions,” is an example of how children engage in personification (Taylor and Mottweiler, 2008). Imaginary companions can either be abstract entities or physical objects, such as dolls and stuffed animals (Singer and Singer, 1990; Taylor and Mottweiler, 2008; Moriguchi and Todo, 2017). Moriguchi and Shinohara (2012) listed examples of imaginary companions; one child reported that “Umechan” is an invisible girl who taught the child everything (Moriguchi and Shinohara, 2012). A famous Japanese movie, *My Neighbor Totoro*, depicts young girls developing friendships with imaginary beings inspired by their natural surroundings. Although these examples are provided by Japanese children, this phenomenon is known to be global (Taylor and Mottweiler, 2008; Lin et al., 2016). Around half of 4-year-old children report having imaginary companions. The number of children who claim to have imaginary companions diminishes gradually as they grow in age from 5- to 12-years-old (Pearson et al., 2001; Boerger et al., 2009; Lin et al., 2016; Moriguchi and Todo, 2017).

This belief in imaginary companions does not mean that children are more credulous than adults nor that they tend to confuse reality with imaginary. Children older than 5 years old are already mature enough to select trustworthy information and doubt testimonies that are counterfactual to their knowledge (Jaswal, 2004; Koenig et al., 2004; Sharon and Woolley, 2004; Boseovski, 2012). Although they often feel strong attachments to them, children are often sure that the imaginary beings are only products of fantasy (Sharon and Woolley, 2004; Taylor and Mottweiler, 2008). Previously, people assumed that the possession of an imaginary companion suggested that the child liked to escape from real situations. However, more recent studies have shown that imaginary companions are healthy phenomena, as demonstrated by findings that there were no significant, negative effects of having imaginary companions on social skills for building relationships with friends or teachers (Manosevitz et al., 1973; Bouldin and Pratt, 1999, 2002; Gleason et al., 2000; Gleason, 2004; Gleason and Kalpidou, 2014). On the contrary, some studies have suggested that having an imaginary companion can serve as a simulation of social exchanges, which enhances the child's understanding of social relationships (Singer and Singer, 1990; Taylor and Carlson, 1997; Taylor and Mottweiler, 2008; Trionfi and Reese, 2009; Gleason and Kalpidou, 2014; Lin et al., 2016).

In this study, we postulated that the tendency of children to engage in personification can be helpful not only in understanding human relationships, but also in understanding relationships between some abstract entities, such as numbers, days, and months. In order to reveal the process by which number personification diminishes throughout development, we focused on measuring the degree to which children engage in personification of numerals zero through nine.

As an index of a child's personification of numbers, we used temporal consistency in the attribution of personality to numbers zero through nine. According to the previous studies on child's personification tendencies, the attribution of personality to non-living objects is not an occasional random guess, but is instead grounded on a strong belief lasting for a certain period of time (Svendsen, 1934; Moriguchi and Shinohara, 2012; Gleason and Kalpidou, 2014; Moriguchi and Todo, 2017). Temporal consistency is a plausible measure to demonstrate that number personification results from a strong belief. We therefore asked participants to represent personality characteristics and tested consistency after 1 month as an index of engagement in number personification.

Prior research has demonstrated that children tend to attribute personality to objects in a personal and idiosyncratic way, as observed in the examples above (Singer and Singer, 1990; Taylor and Mottweiler, 2008; Moriguchi and Shinohara, 2012; Lin et al., 2016; Moriguchi and Todo, 2017). It is likely that the number personification of children is also idiosyncratic, without common agreement across children. Furthermore, if personification worked as a discrimination marker to characterize numbers, each number would be exclusively described by different personality characteristics. We therefore adopted the diversity of personality description as another index to assess number personification.

In a different research context, the personification of numbers has been studied as ordinal linguistic personification (OLP), which is a type of synesthesia. OLP is observable in 1% of the adult population, in which personal traits, such as gender, age, and social roles are assigned to ordinal sequences, including numbers, letters, days, and months (Simner and Hubbard, 2006; Simner and Holenstein, 2007; Smilek et al., 2007; Amin et al., 2011; Sobczak-Edmans and Sagiv, 2013; Simner et al., 2016). OLP is known as a variant of synesthesia (Simner and Hubbard, 2006; Simner and Holenstein, 2007; Smilek et al., 2007; Amin et al., 2011; Sobczak-Edmans and Sagiv, 2013), a condition in which a stimulus (e.g., sound or grapheme) induces an additional experience not commonly associated with the stimulus (e.g., colors) (Baron-Cohen, 1996; Ward, 2008, 2013; Cytowic et al., 2011). Synesthesia is characterized by four basic characteristics, namely, temporal consistency, automaticity, and idiosyncrasy, as well as its low prevalence in an adult population (Ward, 2013), and OLP meets sufficient criteria to be regarded as a variant of synesthesia (Simner and Hubbard, 2006; Simner and Holenstein, 2007; Smilek et al., 2007; Amin et al., 2011; Sobczak-Edmans and Sagiv, 2013).

Number personification in children, which is of the current interest, seems to share several characteristics with OLP. As hypothesized above, number personification in children is expected to be consistent temporally, while the mapping from personality to numbers is idiosyncratic. However, a difference lies in the prevalence; we hypothesized that number personification is a natural extension of a child's general tendency to personify non-living objects and therefore is highly prevalent, whereas OLP is a phenomenon observed in a limited number of adults (Simner and Holenstein, 2007; Smilek et al., 2007; Amin et al., 2011; Sobczak-Edmans and Sagiv, 2013). With respect to prevalence, it should be reasonable to regard number personification in children as a different phenomenon from OLP.

Nevertheless, previous studies of OLP are worth consulting, because OLP has also been assessed through the temporal consistency of the test-retest design. Specifically, we referred to a study of OLP to determine the question items in the questionnaire. Because of the practical considerations in executing child surveys, the questionnaire was designed to have as few questions as possible, and questions were limited to four given characteristics (“gender,” “age,” “goodness,” and “sociability”). The four characteristics were selected by referring to a study that classified typical descriptions of OLP into several subgroups (Smilek et al., 2007). Groups 1-1 and 1-2 represent personal characteristics, while 2-1 and 2-2 denote interpersonal relationships with others, as shown below:

- 1-1) Physical: e.g., gender (*male, female*), age (*late 40s, child*), body shape (*thin, tall, dark hair*)
- 1-2) Personal: e.g., *mischievous, brilliant, serious*
- 2-1) Relational: e.g., *friendly, gets taken advantage of, popular*
- 2-2) Social role: e.g., *younger brother, fatherly, king*

To develop the questionnaire, we selected the four characteristics for the following reasons. First, descriptions of gender and age are the most typical OLP answers (Simner and Holenstein, 2007; Smilek et al., 2007; Amin et al., 2011; Sobczak-Edmans and Sagiv, 2013), and we hypothesized that these would also typically observable in our sample. Although qualitative characteristics of personalities, such as *mischievous* and *self-centred*, vary, we expected that these characteristics could be roughly captured by “goodness.” Although interpersonal descriptions, such as *friendly* and *taking advantage of others*, vary, we assumed that these characteristics could be summarized under “sociability.”

The questionnaire was distributed to 153 child participants in an elementary school, located in Okayama prefecture, Japan (63 fourth graders: 9–10 years old and 90 sixth graders: 11–12 years old). Participants were asked to choose “the most suitable description” regarding each of the four personality characteristics for numbers zero through nine. A similar test was conducted on 55 non-synesthetic adults for comparison purposes. Additionally, we asked participants to conduct number-color mappings and computed their temporal consistency to estimate the prevalence of grapheme-color synesthesia. Since our current research aimed to understand the practice of number personification in non-synesthetic populations, we sought to verify that our sample did not have an unexpectedly higher prevalence of synesthesia than previous works (Simner et al., 2009; Ward, 2013).

## 2. Methods

### 2.1. Participants

A total of 153 Japanese elementary school children participated in the study. Two of them did not complete the questionnaire, so we analyzed the remaining 151 participants' responses (63 fourth graders and 88 sixth graders; mean age = 11.08, *SD* = 1.03). In Japanese elementary schools, fourth graders are 9 to 10 years old, and sixth graders are 11 to 12 years old. To compare the children's results with those of adults, we distributed a similar questionnaire to adults. We recruited 245 undergraduate and graduate students (mean age = 18.9, *SD* = 6.84, 115 males, 130 females). Fifty-five out of the 245 adult participants completed the questionnaire (mean age = 22.6, *SD* = 3.14, 28 males, 27 females).

The study involving adult participants was reviewed and approved by the Ethical Committee of the Department of Arts and Sciences of the University of Tokyo (Project number 222-6). All adult participants provided written informed consent in accordance with the Declaration of Helsinki. The study involving children participants was approved by the Ethical Committee of the Graduate School of Education, Okayama University (Project number 27). Written informed parental consent was obtained for all children participants. All child participants provided informed assent, in which we informed them of the study in a way for children easy to understand. This document was distributed to the children by their teachers in the classroom, where the teachers asked the children to hand the document to their parents (which is a normal way in Japan to distribute some documents from teachers to parents). If the parents consented to their child's participation in the survey, they were asked to write the name of their child on the experimental sheet and post it to our laboratory.

### 2.2. Questionnaire

For child participants, a paper-based questionnaire was conducted, whereas adult participants were asked to complete an Internet-based questionnaire. All procedures were completed in Japanese. The questionnaire was composed of two parts that corresponded to two themes: color and personification. Samples of the questionnaire are shown in Figure S1 online.

The color section assessed the prevalence of grapheme-color synesthesia, which confirmed that our population did not have an unexpectedly higher prevalence of grapheme-color synesthesia compared to the populations used in previous studies (Simner et al., 2009; Ward, 2013). We showed 10 numbers (0–9) in a pseudo-random order, and each number was accompanied by a color palette containing 13 colors (black, dark blue, brown, dark green, gray, pink, purple, orange, red, white, light blue, light green, and yellow; displayed in Figure S1b). We used the same 13 colors in a previous study (Simner et al., 2006), in which the authors developed simple questionnaires for a large-scale survey on grapheme-color synesthesia. We instructed child participants to choose the “most suitable color for each presented number.” We also noted that “there are no right nor wrong answers, so please freely express what you feel.” We asked them to choose one color for each of the numerals and not to repeatedly choose one color. For adult participants, we presented RGB color panels instead of the 13-color palette, with similar instructions (Figure S1c).

The personification section was used to quantify the degree to which participants engaged in number personification. Similar to the color section, the numbers zero through nine were presented in a pseudo-random order and each was followed by four questions (Figure S1a). As summarized in Table 1, four questions were used to describe the four aspects of personification (“gender,” “goodness,” “age,” and “sociability'). Each question had three answers (a pair of antonyms and *none*), which were presented in the order shown in Table 1. Participants were asked to choose the “most suitable description” for the numbers zero through nine. We also noted that “there are no right nor wrong answers, so please freely express what you feel.”

**Table 1:** Questions used to ask about the practice of number.

**Item**	**Answer 1**	**Answer 2**	**Answer 3**
Gender	*Male*	*Female*	*None*
Goodness	*Good*	*Bad*	*None*
Age	*Young*	*Old*	*None*
Sociability	*Having a lot of friends*	*Alone*	*None*

The order of the questions was different between the child and adult questionnaire. For children, the questions on personification were presented first for all 10 numbers (Figure S1a), and the questions on color were presented afterwards (Figure S1b). After 1 month, another questionnaire was distributed as a surprise retest, in which the children were asked to complete at home and return to us within one week. For adults, we asked the personification and color questions at the same time (Figure S1c). Each number (0–9) was accompanied by two questions on color and personification. One month later, we e-mailed participants and requested that they take a surprise retest online. The consistency score of the answers between the first and second questionnaires was computed as an index of grapheme-color synesthesia and number personification.

### 2.3. Data availability

The datasets generated and/or analyzed during the current study are available from the corresponding author upon reasonable request.

## 3. Results

### 3.1. Number-color association to confirm our population was not synesthetic

We assessed the prevalence of grapheme-color synesthesia to ensure that our population did not have an unexpectedly higher prevalence of grapheme-color synesthesia. The temporal consistency between the first and second tests was computed as an index of grapheme-color synesthesia. When a participant chose the same color for one number in both questionnaires, this was scored as 1 point. The summation of all the numbers (0–9) was treated as the participant's consistency score (maximum: 10 points; minimum: 0 points).

To evaluate consistency scores, we consulted a previous work which used the 13-color palette (Simner et al., 2006). In this previous study, the experimenters presented 36 graphemes (26 alphabets and 10 numbers) and asked participants to choose the most suitable color among the 13 colors for each of the graphemes. They computed consistency scores between the first test and the immediate retest. In comparison with the consistency scores given by synesthetes, they determined a rate of 52% consistency as a criterion of synesthesia. Although the work has a different setup from our experiment regarding the number of presented graphemes and the intervals between the first and second tests, their criterion can still be worth referencing and comparing with our result.

**Figure 1:**
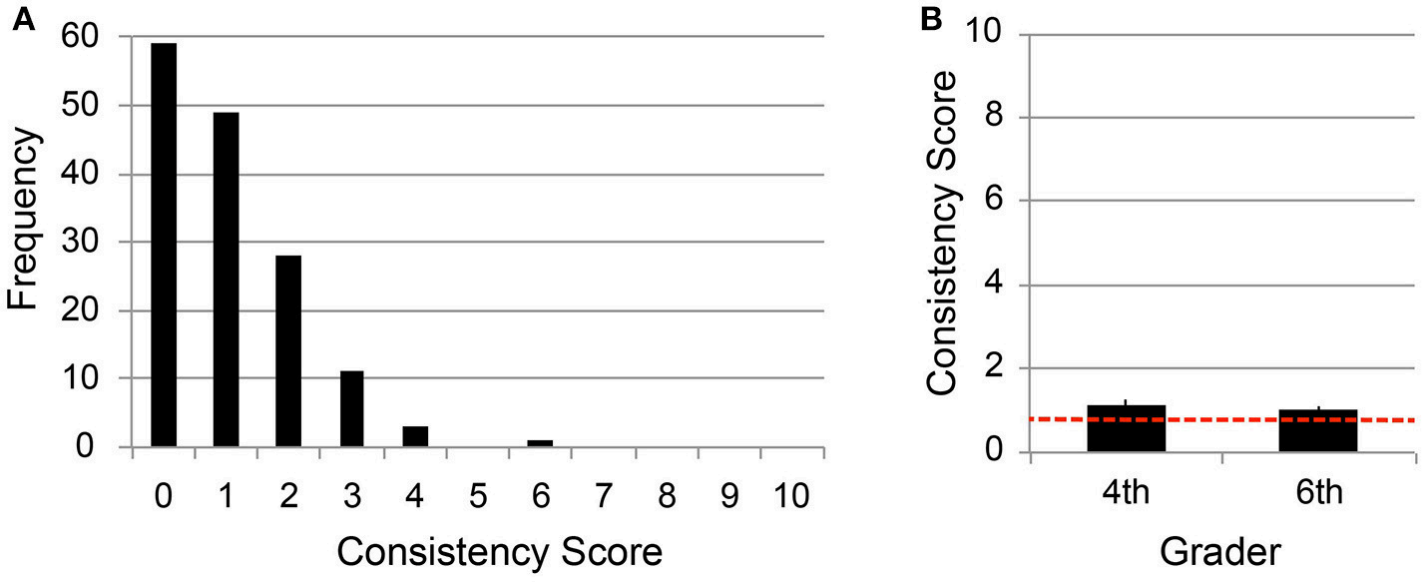
Mean consistency scores of number-color associations. **(A)** Frequency of consistency scores for all the child participants (*N* = 151). **(B)** Mean scores of 4th and 6th graders. Error bars denote *s*.*e*.*m*., whereas the red dotted line indicates chance level (= 0.77).

Figure 1A shows the distribution of the consistency scores of all the child participants. Only one participant out of 151 (= 0.66%) scored more than 52%. The result suggests that our sample did not have an unexpectedly higher prevalence of grapheme-color synesthesia as compared to the study populations in previous works (= 1.4%) (Banissy et al., 2009).

Figure 1B showed the mean consistency scores for the fourth and sixth graders, where the error bars denote standard error of measurement (*s.e.m*.), and the red dotted line indicates the chance level. Chance level was calculated by considering that the probability of choosing the same color twice from the 13 color-palette was 1/13, so the expected score in 10 trials was 0.77 points (1/13·10 = 0.77). No significant difference was found between the mean scores of the fourth and sixth graders [Welch's *T*-test; α = 5%, *t*_(112.6)_ = 0.70, *p* = 0.48]. This finding is consistent with the previous child study(Simner et al., 2006), which found no difference in the level of consistency across ages. A significant difference from the chance level was observed for the fourth graders [one-sample *t*-test, α = 5%, *t*_(62)_ = 2.15, *p* = 0.035], whereas no difference was observed for the six graders [*t*_(87)_ = 1.98, *p* = 0.051 for the sixth graders].

For adults, we used RGB color panels instead of the 13-color palette, which did not allow us to directly compare the results of adults with those of children. We therefore did not use the adults' results for the analyses in this paper. Nevertheless, the results of the child participants already confirmed that the study population did not have an unexpectedly high prevalence of grapheme-color synesthesia, so the results of the adults do not affect the overall conclusion.

### 3.2. Consistency of number personification

The consistency score for number personification was computed from two tests. Similar to the color section, when a participant chose the same answer for a number in both tests, this was scored as “1 point.” However, choosing *none* did not increase the consistency score, even if the answer was consistently *none*. The summation for numbers zero through nine was regarded as the participant's consistency score. The maximum consistency score was 10 points, which was achieved when the participant chose consistent answers (except *none*) for all 10 numbers. Consistency scores were calculated for each of the four characteristic categories (“gender,” “age,” “goodness,” and “sociability”).

Taking the consistency score as an objective variable, a one-way ANOVA was computed by age group (fourth grade, sixth grade, and adults) for each personality factors (“gender,” “goodness,” “age,” and “sociability”). Significant effects were observed for “gender” [α = 5%, *F*(_2,203_) = 4.20, *p* = 0.016], “goodness” [α = 5%, *F*(_2,203_) = 8.52, *p* = 0.0003], “age” [α = 5%, *F*(_2,203_) = 4.67, *p* = 0.010], but not for “sociability” [α = 5%, *F*(_2,203_) = 2.30, *p* = 0.103].

**Figure 2:**
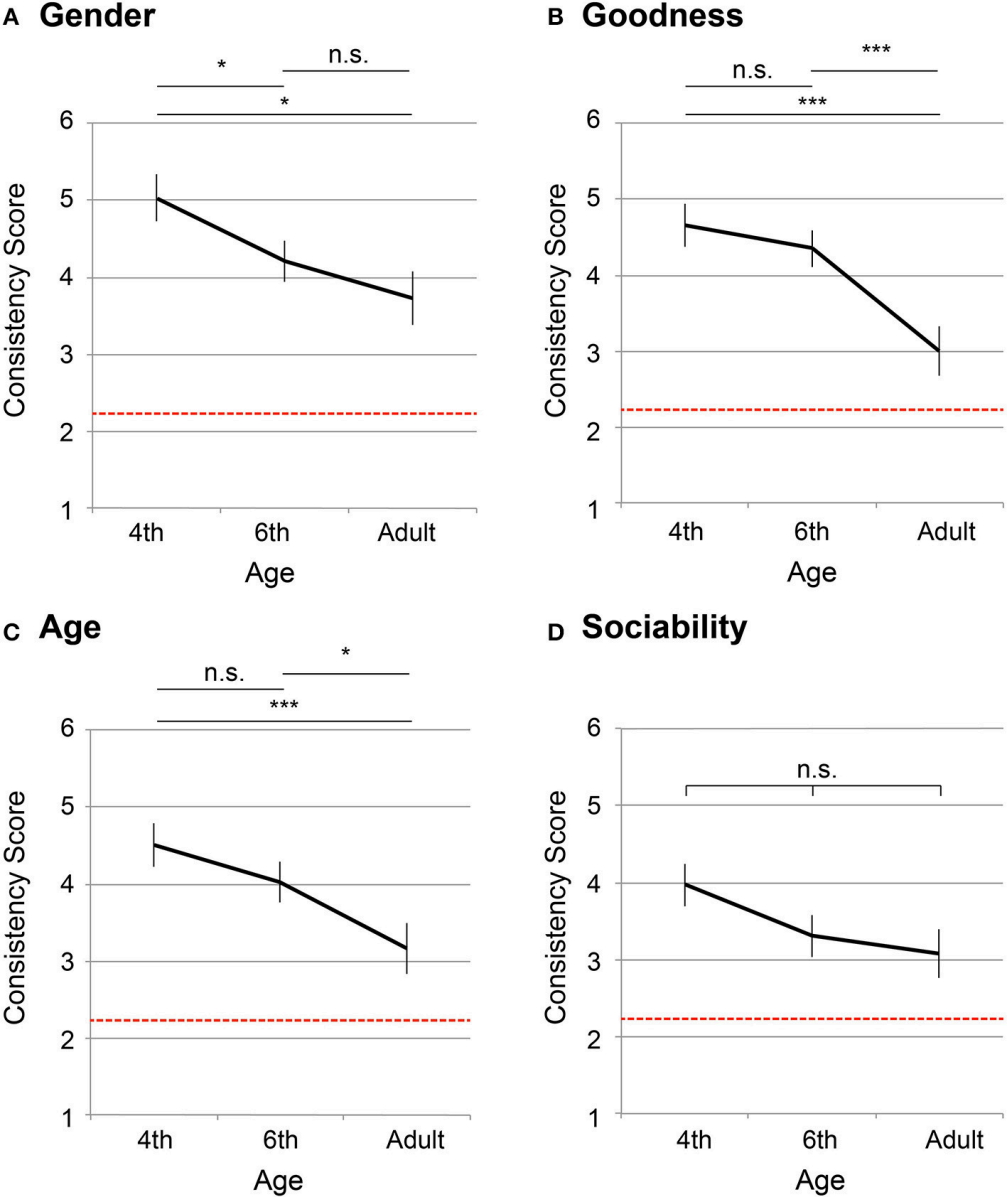
Mean consistency scores of number personification (63 fourth graders, 88 sixth graders, and 55 adults). The x axis shows ages (4th grade, 6th grade, and adults), whereas the y axis denotes the consistency score (maximum: 10 points; minimum: 0 points), averaged across the four personality factors (“gender,” “goodness,” “age,” “sociability”). The error bars denote *s*.*e*.*m*. and the red dotted line indicates chance level (= 2.22). Results of Shaffer's multiple comparisons for the age factor are summarized in^***^p < 0.01, ^*^p < 0.05, n.s. : *p* ≥ 0.05. **(A)** “gender,” **(B)** “goodness,” **(C)** “age,” **(D)** “sociability”.

Figures 2A–D shows the mean consistency scores for each of the personality factors (a: “gender,” b: “goodness,” c: “age,” d: “sociability”). The x axis denotes ages (fourth graders, sixth graders, and adults), and the y axis denotes the mean consistency scores. The error bars in the figure denote *s.e.m*.. The red dotted line indicates the chance level, which was computed as follows: The probability of choosing answers other than *none* on the first test was 2/3, and, on the second test, the probability of choosing the same answer as the first was 1/3. Therefore, the expected consistency score was 2.22 (2/3·1/3·10 = 2.22). Table S1 summarizes Shaffer's multiple comparison between age groups at each of the personality factors, which are depicted in Figure 2A–D by the notification, ^***^: *p* < 0.01, ^*^: *p* < 0.05, n. s. : *p* ≥ 0.05.

### 3.3. Frequency of choosing the *none* option

In the experiment, participants could choose *none* when they did not find any suitable personalities. While the consistency score suggests the degree of number personification, the frequency of choosing *none* can be direct evidence for the disappearance of number personification. We therefore counted the frequency of *none* for each participant (maximum: 10; minimum: 0), to compare across the three age groups. In the analysis on frequency of choosing the *none* option, we considered only the results of the first test and did not consider the results of the second test.

Taking the consistency score for the objective variable, a one-way ANOVA was computed by age group (fourth grade, sixth grade, and adults) for each personality factors (“gender,” “goodness,” “age,” and “sociability”). Significant effects were observed for “gender” [$\alpha =5\%,F{(}_{2,\;203})=9.00,p=0.0002$], “goodness” [$\alpha =5\%,F{(}_{2,\;203})=26.24,p<10^{-4}$], “age” [$\alpha =5\%,F{(}_{2,\;203})=16.26,p<10^{-4}$], and “sociability” [$\alpha =5\%,F{(}_{2,\;203})=9.97,p=0.0001$].

**Figure 3:**
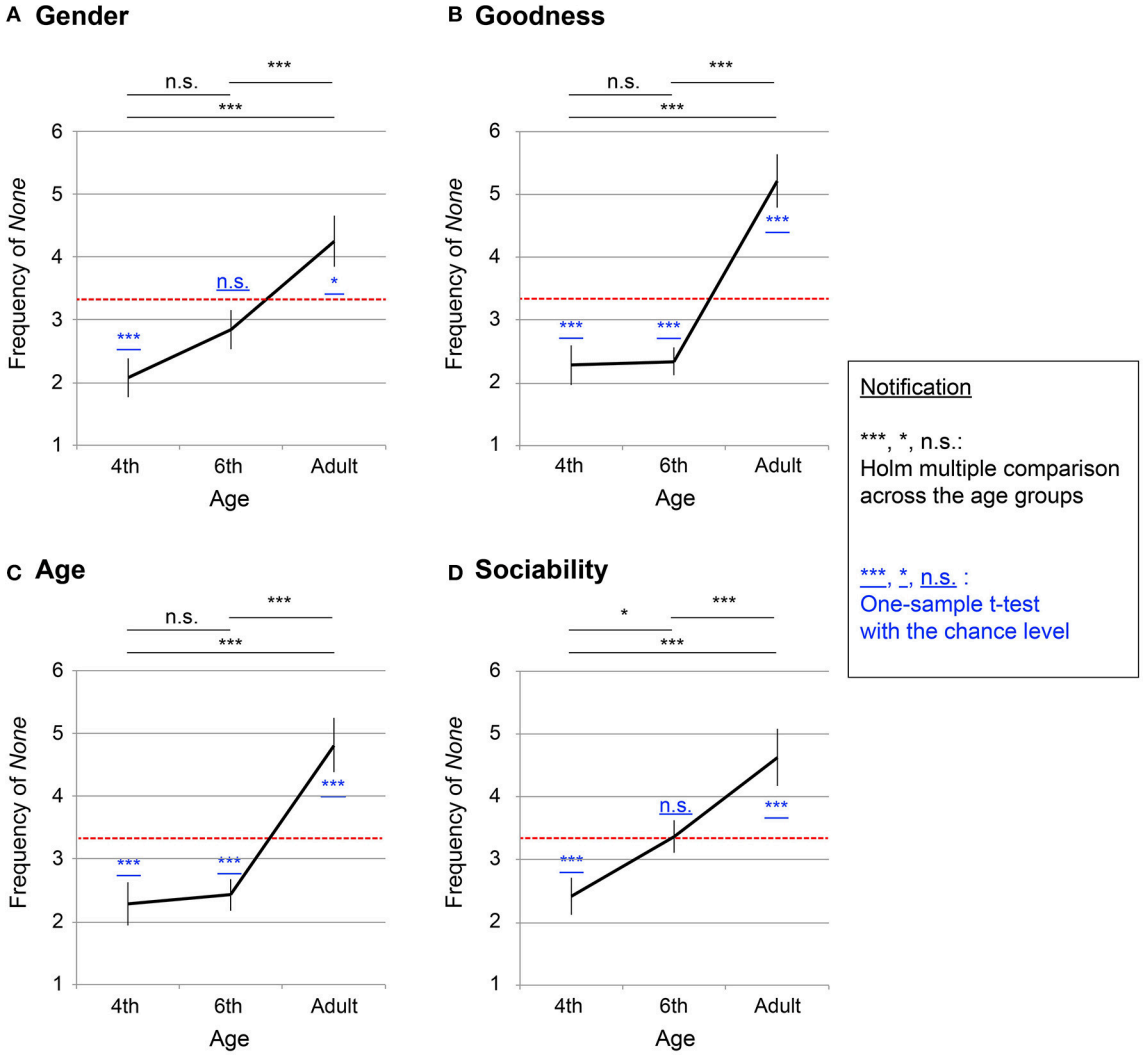
Mean frequency of *none* answers in description of number personification (63 fourth graders, 88 sixth graders, and 55 adults). The x axis shows ages (fourth graders, sixth graders, and adults), while the y axis denotes the mean frequency of *none* (maximum 10 and minimum 0 points). The error bars denote *s*.*e*.*m*. and the red dotted line indicates chance level (= 3.33). Results of Shaffer's multiple comparison between age groups are summarized by black-colored symbols: ^***^p < 0.01; ^*^p < 0.05; and n.s. : *p* ≥ 0.05. The result of one-sample *t*-test with a chance level summarized by symbols underlined in blue: ^***^p < 0.01; ^*^p < 0.05; and n.s. : *p* ≥ 0.05. **(A)** “gender,” **(B)** “goodness,” **(C)** “age,” **(D)** “sociability”.

Figures 3A–D shows the mean frequency of *none* for each of the personality factors (a: “gender,” b: “goodness,” c: “age,” d: “sociability”). The *x* axis denotes ages (fourth graders, sixth graders, and adults), and the *y* axis indicates the mean frequency of *none*. The error bars denote *s*.*e*.*m*., whereas the red dotted line indicates the chance level (= 3.33). The chance level was calculated as follows: Given a participant randomly chose one option among three (e.g., *male, female, none*), he/she would choose *none* with one-third probability. Therefore, the expected frequency of answering *none* for the numbers 0–9 became 3.33 (1/3·10 = 3.33). Table S2 summarizes Shaffer's multiple comparison between the age groups at each of the personality factors, which are depicted in Figures 3A–D by the notification, ^***^: *p* < 0.01, ^*^: *p* < 0.05, n. s. : *p* ≥ 0.05.

A one-sample *t*-test was conducted to compare the frequency of *none* with the chance level (= 3.33; summarized by the notification underlined in blue in Figure 3, ^***^: *p* < 0.01,^*^: *p* < 0.05, n.s.: *p* ≥ 0.05). The detailed statistical variables including *t*-values, degrees of freedom, and *p*-values, are summarized in Table S3. The fourth-grade children chose *none* significantly less than chance (*p* < 0.05 for all four personality factors), whereas the adults chose *none* significantly more (p < 0.05 for the all four factors). The sixth-grade children took middle values between the fourth graders and the adults: For “goodness” and “age” (*p* < 0.05), the sixth-grade children chose *none* significantly less than chance, whereas, for “gender” and “sociability,” they did not show any significant differences [*t*_(87)_ = −1.58, *p* = 0.12 for “gender”; *t*_(87)_ = 0.17, *p* = 0.86 for “sociability”]. This result suggests that the personification traits of “gender” and “sociability” diminish earlier than “goodness” and “age,” which is discussed later in the discussion section.

### 3.4. Diversity of description

The results indicate that some participants attributed diverse personalities to each number, whereas some repeatedly assigned similar descriptions to all 10 numbers. For instance, one participant gave various personalities to the numbers; the participant indicated that the number “2” is *female, good, young*, and *having a lot of friends*, and the number “5” is *male, bad, none*, and *alone*. On the other hand, some repeatedly used the descriptions of *male, good, young*, and *having a lot of friends* for most of the 10 numbers, while some chose only *none*. We considered that the diversity of description can be another measurement to assess the degree of number personification. In actual human relationships, individuals are characterized by their unique personalities, so the description of personality should be as diverse as the number of people. Likewise, if a participant treated numbers as if they truly had personalities, the participant would assign unique characteristics to each numbers.

To quantify the diversity, we counted the number of different personality combinations attributed to the 10 numbers by each participant. For simplicity, we wrote a combination of a personality in a vector form (*x*^*gender*^, *x*^*goodness*^, *x*^*age*^, *x*^*sociability*^), which we called a “personality vector”. Let a value of *x*^*i*^ be an element of the personality vector; namely, *x*^*i*^ took either *x*^*gender*^, *x*^*goodness*^, *x*^*age*^, or *x*^*sociability*^. We defined *x*^*i*^ = 1 when answer 1 (in Table 1) was chosen consistently in the first and second tests (i.e., *male, good, young*, or *having a lot of friends*), *x*^*i*^ = −1 when answer 2 was chosen consistently (i.e., *female, bad, old*, or *alone*), and *x*^*i*^ = 0 when answer 3 (i.e., *none*) was chosen consistently or any answers 1–3 were chosen inconsistently. We regarded *none* and inconsistent answers as being in the same category. For instance, the description of (*female, good, young, having a lot of friends*) is rewritten by (–1, 1, 1, 1), and (*male, bad, no age, alone*) takes (1, –1, 0, –1). We then counted the number of different vectors for each participant. If the participant assigned different vectors to all 10 numbers, then he/she received the maximum 10 points. If the participant repeatedly chose similar or inconsistent answers, then he/she obtained fewer points.

**Figure 4:**
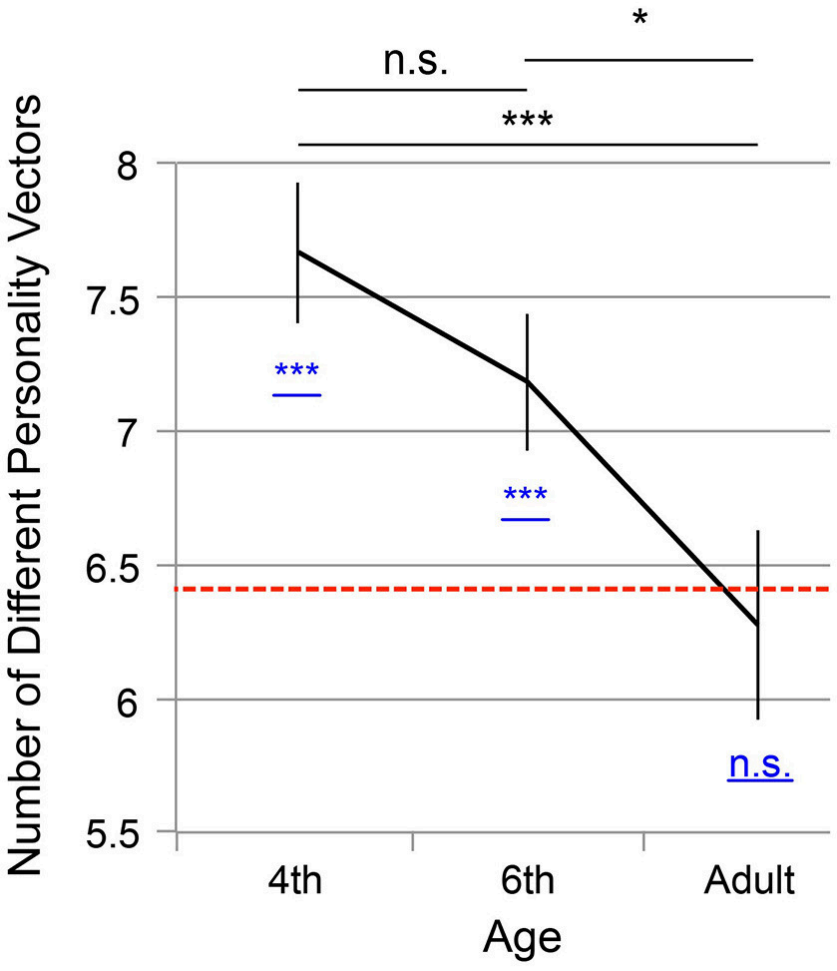
Mean number of personality descriptions (maximum: 10; minimum: 1) for the three age groups: 4th graders (*N* = 63), 6th graders (*N* = 89), and adults (*N* = 55). Error bars denote *s.e.m*. and the red dotted bar indicates an estimated chance level by computer simulation (= 6.41). The results of Shaffer's multiple comparison are summarized by the black symbols: ^***^p < 0.01; ^*^p < 0.05; and n.s. : *p* ≥ 0.05). The result of the one-sample *t*-test with the chance level is depicted by symbols underlined in blue: ^***^p < 0.01 and n.s. : *p* ≥ 0.05.

Figure 4 shows the number of the personality vectors averaged across participants. The *x* axis denotes the age groups (fourth graders, sixth graders, and adults), and the *y* axis indicates the average number of the personality vectors. The error bars indicate *s.e.m*. and the red dotted bar depicts the chance level (= 6.41).

The chance level was difficult to solve analytically, so we estimated it with a computer simulation. Let us consider a virtual participant who is implemented in a computer simulation and answers in a random way. The probability of *x*^*i*^ = 1 (denoted by *P*(*x*^*i*^ = 1)) is 1/3·1/3 = 1/9, *P*(*x*^*i*^ = −1) = 1/3·1/3 = 1/9, and *P*(*x*^*i*^ = 0) = 1−*P*(*x*^*i*^ = 1)−*P*(*x*^*i*^ = −1) = 1−1/9−1/9 = 7/9. At these probabilities, the virtual participant receives 10 personality vectors that correspond to the numbers zero through nine. We repeated the process and created 100,000 virtual participants for each of whom we counted the number of different combinations of personality vectors in the same way we did for the actual participants (as explained above). We found that the number of personality vectors converged to 6.41, which we therefore adopted as an estimated chance level.

As the result, a significant decrease in the number of personality vectors was observed across the fourth graders, sixth graders, and adults [one-way ANOVA, α = 5%, *F*(_2,203_) = 5.25, *p* = 0.0060]. Shaffer's multiple comparison showed significant decreases between fourth graders and the adults [α = 5%, *t*_(_203) = 3.20, *p* = 0.0048] and between sixth graders and the adults [*t*_(203)_ = 2.24, *p* = 0.026] but no significant difference between the fourth and sixth graders [*t*_(203)_ = 1.25, *p* = 0.21]. For comparison with the chance level, a one-sample t-test was conducted. The fourth and sixth graders showed significantly higher scores than chance [α = 5%, *t*_(62)_ = 4.78, *p* < 10^−4^ for the fourth grader and *t*_(87)_ = 3.04, *p* = 0.0031 for the six grader], but scores of the adults were not significantly different from the chance [*t*_(54)_ = 0.39, *p* = 0.70]. This result suggests that younger children tend to attribute diverse characteristics to the numbers zero through nine, and this diversity tends to diminish with development.

## 4. Discussion

In the present study, we examined the tendency of non-synesthetic, elementary school children to engage in number personification. We observed that number personification changed throughout development, with respect to temporal consistency, frequency of number personification, and the diversity of personality descriptions. While most of our analyses did not show significant differences between the fourth graders and the sixth graders, the mean values of the fourth graders in all three analyses suggested that this group had a stronger tendency to engage in number personification than the sixth graders (Figures 2–4). On the other hand, there were significant differences between the children—both fourth and sixth graders—and adults.

The consistency score in the test-retest design may represent better memory performance, rather than the strength of association. If the higher consistency score resulted from better memory performance, then participants who achieved higher consistency scores in the number-personality mapping would have shown higher scores also in the number-color mapping. As depicted in Figure 1, 2, however, the 4th grade children exhibited higher consistency scores specifically in number personification, whereas, in the case of number-color mappings, the score of 4th grade children were not significantly different from that of the 6th graders. This finding suggests that higher consistency does not mean better memory capability.

Our target years of age (9–12 years old) were in the middle of an important developmental change: the movement from the “concrete” operational stage to the “formal” operational stage (Piaget, 1954a,b). In the concrete operational stage (around 7–12 years old), children are not considered good at operating purely abstract concepts, such as algebra equations. At these ages, children use concrete objects as analogies for abstract concepts, which can help them to understand abstract concepts. Metaphorical expressions are often used as such analogies. For instance, in a case study, a child reported that “I *stand back* a number” and “I *started at* twenty and counted *along* to sixty”(Bills, 2003; Núñez, 2004), in which children used metaphors originated from their embodied cognitions as analogies for number alignments. In the formal operational stage (around 12 years old and over), children become able to think abstractly and reason hypothetically without any concrete images (Piaget, 1954a,b).

For our child participants, especially the fourth graders, number personification could function as concrete imaginations to help their mathematical operations. Some of the participants in the sixth grade may have been moving to the formal operational stage, so their number personification was becoming weaker. For adults, number personification became even less necessary and, therefore, diminished. The decrease in the diversity of number personification supports this view. As was seen in our child samples, the more diverse personalities a participant gives to numbers, the more clues he/she will obtain to discriminate numbers. On the other hand, as observed in the adult sample, assignment of homogeneous personalities suggests less functionality of number personification as a discrimination marker.

A question arises of whether the number personification observed in this paper is also a type of metaphor (like the above examples) or something else. In metaphorical expressions, people within the same culture normally agree on common descriptions (Deroy and Spence, 2013). For instance, many people in Western cultures regard numbers as points on a line (Lakoff and Núñez, 2000; Núñez et al., 2012). People agree that continuity is gapless, whereas recurrence is circular. However, in our experiment, participants attributed various descriptions to numbers, and no outstanding tendencies were observed. Some participants assigned *male* to number “2”; some described “2” as *female*; and some chose *none* (Figure S2). As noted in the introduction, the idiosyncrasy of a child's number personification is consistent with the general personification of children (Singer and Singer, 1990; Taylor and Mottweiler, 2008; Moriguchi and Shinohara, 2012; Lin et al., 2016; Moriguchi and Todo, 2017). With respect to idiosyncrasy, the number personification of children is discriminated from the metaphorical expression of mathematics.

It is beyond the scope of the current study to prove to what extent number personification in children is similar to adults' OLP. However, the number personification of children appears to have share some basic characteristics with OLP. The temporal consistency and idiosyncrasy were shared both by the number personification of children and by OLP among synesthetes (Smilek et al., 2007; Deroy and Spence, 2013). A critical difference lies in the prevalence; while the number personification was prevalent in a normal child population, OLP is known to be observed in a limited number of adult population (Simner and Hubbard, 2006; Simner and Holenstein, 2007; Smilek et al., 2007; Amin et al., 2011; Sobczak-Edmans and Sagiv, 2013; Simner et al., 2016). A possible hypothesis could be drawn; OLP can have a origin similar to the general personification of children and therefore share similar characteristics. Namely, number personification normally diminishes during development, but a few of adults retain the tendency, which can be recognized as OLP of adults. Indeed, it is consistent with the pruning hypothesis (Rich and Mattingley, 2002; Maurer and Mondloch, 2005), in which synesthesia, including OLP, exists commonly in early childhood and becomes diminished through synaptic pruning. On the other hand, the consequence would contradict a previous child study on grapheme-color synesthesia (Simner et al., 2009). The study suggested a similar level of prevalence of grapheme-color synesthesia in child and adult populations (1%). The contradiction could be an implication that grapheme- color synesthesia and OLP would be classified into different types of synesthesia. Further research on OLP in children is warranted to reveal the relationship between number personification and OLP. Next, we would like to consider the process by which number personification disappears with increasing age. Figures 2, 4 show no significant differences between the fourth and sixth graders, whereas there were significant differences both between the fourth graders and adults and between the sixth graders and adults. However, in Figure 3D, the sixth graders chose *none* significantly more often than the fourth graders did for the “sociability” trait, whereas they did not do the same for the other three traits (“gender,” “age,” and “goodness”) (Figures 3A–C). This finding suggests that the sociability trait vanishes earlier than the other three personality factors. As discussed in the introduction of this paper, descriptions of OLP can be classified into either personal or interpersonal sub-groups, in which synesthetes behave differently (Smilek et al., 2007). Among our four personality questions, “sociability” is categorized into the former and the other three belong to the latter. Interpersonal characteristics seem to be more helpful for understanding mathematics because mathematics concerns relationships between numbers, rather than the individual characteristics of numbers. Thus, the number personification observed in the sixth graders lacked the essential element as a discrimination marker. This result could be because the sixth graders, who are just at the edge between the concrete and operational stages, did not actually require number personification to support mathematical operations.

In this study, it was difficult to detect any regularities or biases in mapping between numbers and personalities. Figure S2 summarizes the ratios between Answers 1 and 2 depicted in Table 1. For the numbers “7” and “9,” there were no significant biases between *male* and *female* (chi-squared test), whereas *male* was attributed more frequently to the numbers “1” and “5.” The answer *bad* was often chosen for the numbers “4” and “9.” This is consistent with the Japanese language, where the pronunciation of the number “4” (*si*) is the same as that of “death,” and the sound of the number “9” (*ku*) means suffering, meaning that the numbers “4” and “9” are symbols of misfortune. This finding suggests that cultural factors affect number personification.

A possible explanation for the failure to find any regularities in the association between numbers and personalities is that we examined only 10 numbers with three discrete options. The number of graphemes examined in this study is relatively small compared to that of previous studies that found some regularities in mappings from graphemes (and numbers) to colors (Watson et al., 2012; Asano and Yokosawa, 2013). Those previous studies examined all 26 letters of the alphabet and 10 numbers using continuous RGB values. To reveal regularities behind the association between numbers and personalities, it would be useful to examine children's personification of other number-related entities, such as days and months. Another possible explanation for the failure to capture regularities is that number personification is essentially idiosyncratic. As discussed in the introduction section, children create imaginary companions that are idiosyncratic and original, and foster special friendships with these companions. Likewise, children may attribute personalities to numbers to build special friendships with them. If this is true, the four personality descriptions given in the current experiment are not enough to describe children's personification of numbers. In future research, it would be useful to focus on descriptive features of number personification to comprehensively describe children's number personification.

## 5. Author contributions

EM and YO contributed design of the study and performed the experiments. EM performed the statistical analysis. EM, MA, and KY interpreted the data. EM wrote the first draft of the manuscript. MA and KY directed the research goal and final conclusion. All authors contributed to manuscript revision, read, and approved the submitted version.

### Conflict of interest statement

The authors declare that the research was conducted in the absence of any commercial or financial relationships that could be construed as a potential conflict of interest.
