# Supplementary material for: Developmental Changes in Number Personification by Elementary School Children
Source: Front Psychol. 2018 Nov 15;9:2214. doi: 10.3389/fpsyg.2018.02214 (PMC6249874; doi:10.3389/fpsyg.2018.02214)
Supplement: Supplementary file 1 [file Table_1.PDF]

Table S1. Consistency scores for each of the personality factors: The result of Shaffer's multiple comparison for participant age groups. The symbols summarize the results as ***: $p < 0.01$; *: $p < 0.05$; and n.s.: $p \geq 0.05$).

Pair	t-value	d.f.	adjusted p-value	
**Gender**				
4th – 6th	2.01	203	0.046	*
4th – Adults	2.83	203	0.015	*
6th – Adults	1.11	203	0.27	n.s.
**Goodness**				
4th – 6th	0.78	203	0.44	n.s.
4th – Adults	3.87	203	0.0004	***
6th – Adults	3.40	203	0.0008	***
**Age**				
4th – 6th	1.22	203	0.22	n.s.
4th – Adults	3.03	203	0.0084	***
6th – Adults	2.08	203	0.039	*
**Sociability**				
-				
