# Supplementary material for: Developmental Changes in Number Personification by Elementary School Children
Source: Front Psychol. 2018 Nov 15;9:2214. doi: 10.3389/fpsyg.2018.02214 (PMC6249874; doi:10.3389/fpsyg.2018.02214)
Supplement: Supplementary file 2 [file Table_2.PDF]

Table S2. Frequency of choosing the *none* option: The result of Shaffer's multiple comparison for the age factor at each of the personality factors. The symbols summarize the results as ***: $p < 0.01$; *: $p < 0.05$; and n.s.: $p \geq 0.05$).

Pair	t-value	d.f.	adjusted p-value	
**Gender**				
4th – 6th	1.64	203	0.102	n.s.
4th – Adults	4.19	203	$< 10^{-4}$	***
6th – Adults	2.92	203	0.0038	***
**Goodness**				
4th – 6th	0.13	203	0.90	n.s.
4th – Adults	6.25	203	$< 10^{-4}$	***
6th – Adults	6.58	203	$< 10^{-4}$	***
**Age**				
4th – 6th	0.32	203	0.75	n.s.
4th – Adults	5.03	203	$< 10^{-4}$	***
6th – Adults	5.09	203	$< 10^{-4}$	***
**Sociability**				
4th – 6th	2.16	203	0.032	*
4th – Adults	4.46	203	$< 10^{-4}$	***
6th – Adults	2.72	203	0.0072	***
