# Supplementary material for: Developmental Changes in Number Personification by Elementary School Children
Source: Front Psychol. 2018 Nov 15;9:2214. doi: 10.3389/fpsyg.2018.02214 (PMC6249874; doi:10.3389/fpsyg.2018.02214)
Supplement: Supplementary file 3 [file Table_3.PDF]

Table S3. Statistical variables of one-sample t-test to compare the frequency of *none* with the chance level (=3.33). The symbols summarize the results as ***: $p < 0.01$; *: $p < 0.05$; and n.s.: $p \geq 0.05$).

Age group	t-value	d.f.	p-value	
**Gender**				
4th	-4.00	62	0.00018	***
6th	-1.58	87	0.12	n.s.
Adults	2.28	54	0.027	*
**Goodness**				
4th	-3.27	62	0.0018	***
6th	-4.41	87	$< 10^{-4}$	***
Adults	4.33	54	$< 10^{-4}$	***
**Age**				
4th	-3.01	62	0.0037	***
6th	-3.53	87	0.00067	***
Adults	3.40	54	0.0011	***
**Sociability**				
4th	-3.13	62	0.0027	***
6th	0.17	87	0.86	n.s.
Adults	2.81	54	0.0061	***
