# Supplementary material for: Developmental Changes in Number Personification by Elementary School Children
Source: Front Psychol. 2018 Nov 15;9:2214. doi: 10.3389/fpsyg.2018.02214 (PMC6249874; doi:10.3389/fpsyg.2018.02214)
Supplement: Supplementary file 4 [file Image_1.PDF]

Supplementary Information

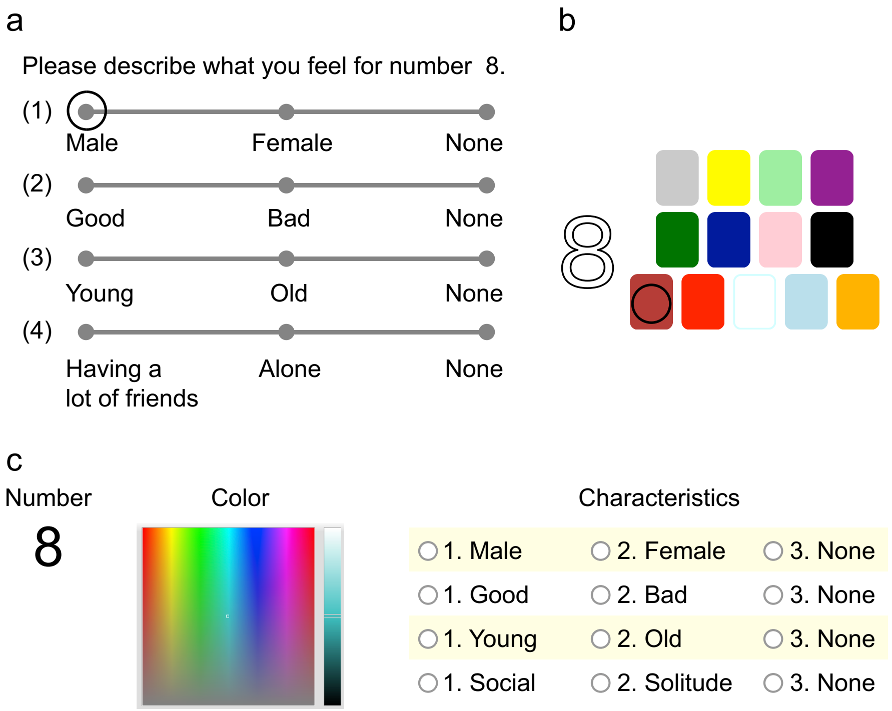

Figure S1. Appearance of the questionnaire. a & b: Paper-based test for the child study, where a: items ask personality, and b: items ask colour of the number '8'. c: Computer interface for the adult study. Numbers 0–9 were presented in a randomized order.
