# Supplementary material for: Developmental Changes in Number Personification by Elementary School Children
Source: Front Psychol. 2018 Nov 15;9:2214. doi: 10.3389/fpsyg.2018.02214 (PMC6249874; doi:10.3389/fpsyg.2018.02214)
Supplement: Supplementary file 5 [file Image_2.PDF]

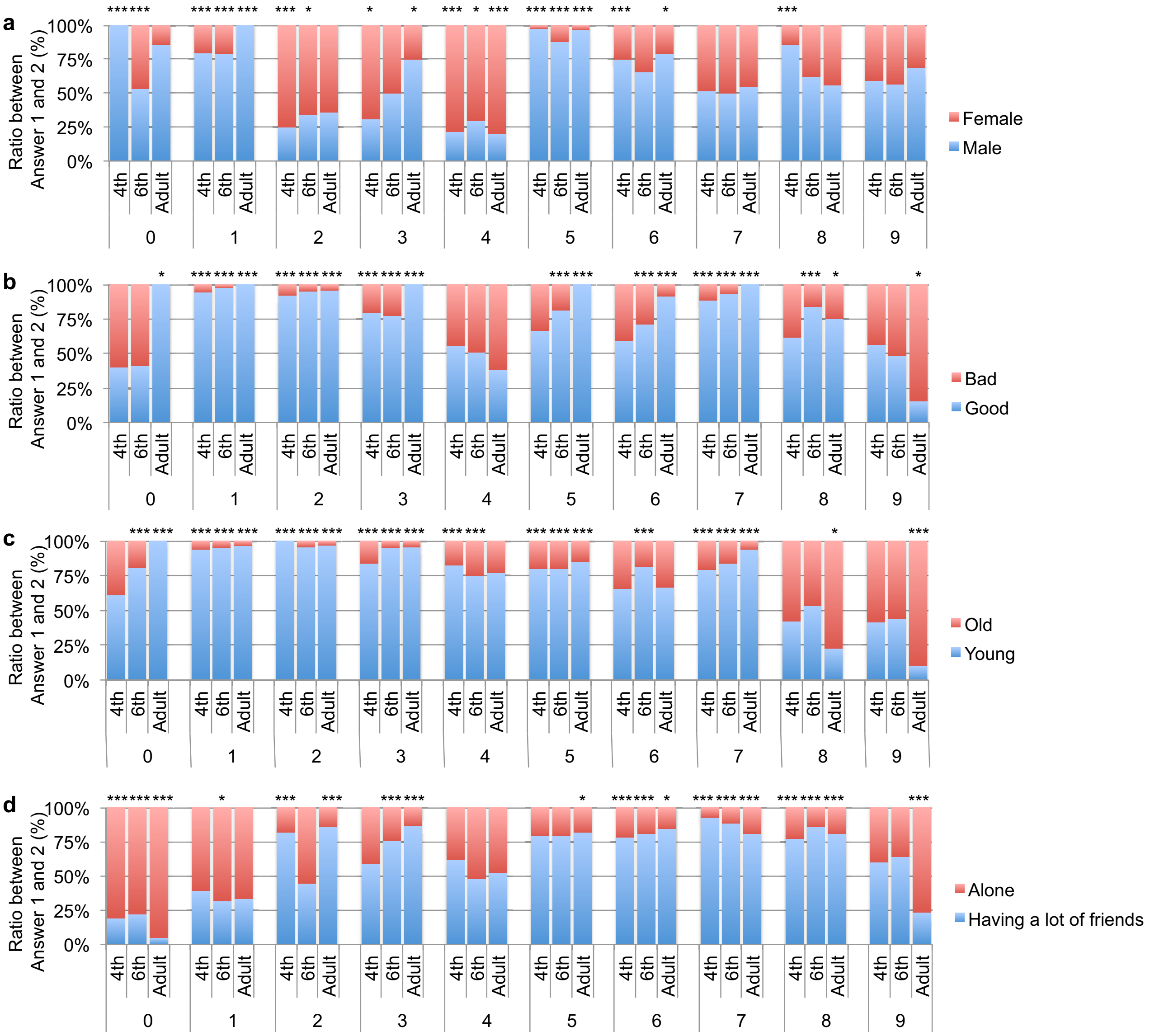

Figure S2. Attribution of personalities. The x axis represents number 0–9 for the three respective age groups, and the y axis indicates the ratio between Answer 1 versus Answer 2 listed in Table 1. Only consistent answers were counted. a: gender, b: goodness, c: age, d: sociability. The blue- and red-coloured bars (blue or red) correspond to Answers 1 and 2, respectively. Significant biases between Answers 1 and 2 were denoted by the mark * (chi-squared test, ***: $p < 0.01$; and *: $p < 0.05$).
